# Aerial Conveyance of Infectious Diseases

**Published:** 1914-07-04

**Authors:** 


### AERIAL CONVEYANCE OF INFECTIOUS DISEASES.

### A Hint for Fever Hospital Administrators.

For a long time a good many members of an expert group of institutional specialists—namely, the medical officers of infectious fever hospitals—have been losing faith in the once generally accepted doctrines of the method of spread of these diseases by aerial conveyance. For the most part this change of view has been based on accumulated clinical experience not easily capable of translation into statistics and figures—that is, not easily capable of anything in the nature of exact proof.

A valuable contribution by Dr. F. H. Thomson and Mr. C. Price to the study of the method of spread of fevers has appeared in the *Lancet*. These observers have for two years carried out most carefully devised trials of a system of bed isolation, in which all sorts of infectious fevers were admitted into one and the same ward. The very strictest precautions were taken by the provision of separate utensils for each case to exclude all possible sources of cross-infection from one case to another, except that of aerial convection. At each bedside hung a special gown to be worn by anyone attending or touching the patient in that bed. Careful sterilisation of everything that might come in contact with more than one patient was carried out; and as an instance of the thoroughness of the precautions taken to make the experiment scientifically useful, even a separate pen was provided for the necessary prescribing, charting, and so on, connected with each separate case. Picked sisters and nurses were employed in the ward, all fully trained in the minutiæ of the bed-isolation system adopted. No patient was allowed out of bed on any pretext, and everything that forethought could dictate was done to exclude every source of infection except such as might circulate in the air of the (freely ventilated) ward.

The authors of this research are properly cautious about their deductions and tentative in their conclusions. Among other important points which they have learnt from their experience, they lay stress on the stage at which the case of infectious disease has arrived as influencing the possibility of diffusible infection. No deductions, they find, can safely be drawn from lists of different infectious diseases treated in the same ward unless the ages of the patients and the day of development of their diseases are clearly stated. So, too, it is very necessary that regard should be paid to the state of protection (by previous attack or otherwise) of the other patients in the ward to any particular disease of which a case happens to be admitted.

Taken as a whole, Dr. Thomson and Mr. Price are clearly unfavourable to the aerial conveyance hypotheses, with certain limitations. If their experiments and their views are accepted, both hospital architecture and hospital administration may be materially influenced in the future. They feel that they can treat infectious diseases in one ward, almost certainly, with perfect safety if they could select the day of the disease on which to admit them. They are satisfied that the treatment of the above diseases is more satisfactorily carried out in this class of ward than in a cubicle ward—that is, in rooms with incomplete partitions between the beds. The supreme advantage of the open ward, as has long ago been pointed out in The Hospital, is the much greater degree of supervision that is possible. The sister, or the charge nurse, can see everything that goes on in the ward; unless the nursing staff can do this, isolation measures and ward discipline cannot be ensured, whether the patients be adults or children.

On the subject of box wards, Dr. Thomson and Mr. Price are much less emphatic. For the treatment of diseases which are not conveyed by air an open ward, they say, is possibly better than one with complete partitions. Here, we think, they display some confusion of thought. For their object is to discover whether or not aerial conveyance of infection is possible; and they are not entitled to assume that these diseases are not capable of being air-borne in discussing the relative advantages of bed-isolation in open wards and in box wards. In the case of scarlet fever they think the evidence from their experiment goes strongly to show that the infection is not air-borne. The evidence about whooping-cough is less definite, and they are prepared to admit that it may be occasionally air-borne. Measles may be transmitted through the air early in the disease, but this capability is soon lost. Of chicken-pox much the same view is taken: on and after the third day it is probably not air-borne, before that it may be. Diphtheria they regard as not air-borne at all. Of German measles and mumps no air-borne case of cross-infection arose; but the small number of cases observed scarcely warrants any definite conclusion on these diseases. Still, previous experience of the authors, combined with the results in this experimental ward, inclines them to the belief that neither of these fevers is air-borne.

These interesting researches were conducted in the North-Eastern Fever Hospital, Tottenham. There is no doubt that they will excite much discussion among other fever hospital medical officers; and it is most desirable that further independent investigations should be undertaken to test the conclusions now expressed. If these should be confirmed, institutional practice may be profoundly modified as a result. There is one respect in which the nursing profession also may be affected. The description of the technique which had to be elaborated in order to close every possible loophole for the conveyance of infection from one patient to another during the necessary handling and other nursing attendance, shows that an exceedingly high standard of training was reached by those to whom the ward was entrusted. Without any disparagement of fever nurses as a body, it is only plain truth to say that the general average of technical efficiency is less high, and that it will be hard work for all concerned to attain the higher level demanded by the open ward and mixed case system.

